# Chronic Activation of Hepatic Nrf2 Has No Major Effect on Fatty Acid and Glucose Metabolism in Adult Mice

**DOI:** 10.1371/journal.pone.0166110

**Published:** 2016-11-04

**Authors:** Sebastian Brachs, Angelika F. Winkel, James Polack, Hui Tang, Maria Brachs, Daniel Margerie, Bodo Brunner, Kerstin Jahn-Hofmann, Hartmut Ruetten, Joachim Spranger, Dieter Schmoll

**Affiliations:** 1 Department of Endocrinology, Diabetes and Nutrition, Center for Cardiovascular Research, Charité –University School of Medicine, Berlin, Germany; 2 German Center for Cardiovascular Research, DZHK Partner site Berlin, Berlin, Germany; 3 Sanofi-Aventis Deutschland GmbH, Industriepark Hoechst, Frankfurt am Main, Germany; East Tennessee State University, UNITED STATES

## Abstract

The transcription factor NF-E2-related factor 2 (Nrf2) induces cytoprotective genes, but has also been linked to the regulation of hepatic energy metabolism. In order to assess the pharmacological potential of hepatic Nrf2 activation in metabolic disease, Nrf2 was activated over 7 weeks in mice on Western diet using two different siRNAs against kelch-like ECH-associated protein 1 (Keap1), the inhibitory protein of Nrf2. Whole genome expression analysis followed by pathway analysis demonstrated successful knock-down of Keap1 expression and induction of Nrf2-dependent genes involved in anti-oxidative stress defense and biotransformation, proving the activation of Nrf2 by the siRNAs against Keap1. Neither the expression of fatty acid- nor carbohydrate-handling proteins was regulated by Keap1 knock-down. Metabolic profiling of the animals did also not show effects on plasma and hepatic lipids, energy expenditure or glucose tolerance. The data indicate that hepatic Keap1/Nrf2 is not a major regulator of glucose or lipid metabolism in mice.

## Introduction

The transcription factor NF-E2-related factor 2 (Nrf2) regulates the expression of several cytoprotective genes and is a promising target for the treatment of chronic, oxidative stress–related disorders, such as neurodegenerative diseases and chronic kidney disease [1–3]. Nrf2 is regulated by the kelch-like ECH-associated protein 1 (Keap1). Keap1 acts as a sensor for oxidative and electrophilic stress. Without stressors, Keap1 mediates the ubiquitinylation and subsequent proteolytic degradation of Nrf2. In the presence of oxidative or electrophilic stress, cysteine residues within Keap1 become modified, which prevents the degradation of Nrf2. Nrf2 subsequently accumulates in the nucleus, where it regulates the expression of several antioxidative and anti-inflammatory proteins by binding to the cis-acting antioxidant response element within gene promoters.

Nrf2 activation has also been linked to the regulation of intermediary metabolism and could therefore be a promising approach for the treatment of steatosis, as well as insulin resistance. [3–5]. Evidence for this derives from studies using pharmacological activators of Nrf2 such as CDDO-Im, CDDO-Me and Oltipraz. Here, a reduction of obesity and hepatic lipids and an improvement of glucose tolerance were observed in rodent models [6–8]. However, these molecules are associated with toxic effects, which could have been a considerable confounding factor in the metabolic studies [9, 10]. Genetic models leading to either a loss-of- or gain-of-Nrf2 function gave no clear picture about the role of Nrf2 in hepatic lipid and carbohydrate metabolism [11–20]. With that it is unclear, whether the activation of Nrf2 has a reasonable therapeutic potential to prevent dysregulation of lipid and carbohydrate metabolism under unfavorable environmental conditions as reflected by a Western diet. In the present study we assessed the effects of inducible Nrf2 activation in the hepatic metabolism of adult mice under a Western diet. Due to the lack of suitable pharmacological tool compounds [21], we activated Nrf2 in liver using siRNAs against Keap1 as recently described [22]. Our data suggest that the chronic activation of Nrf2 in liver has no major effect on metabolism under the challenge of a Western diet.

## Material and Methods

### Reagents and chemicals

All chemicals were purchased from Sigma-Aldrich (Munich, Germany), Merck (Darmstadt, Germany) or Roth (Karlsruhe, Germany), reagents for RNA, cDNA and qPCR from Thermo Scientific (Schwerte, Germany) or Qiagen (Hilden, Germany) unless stated otherwise.

### Animal studies

All animal experiments were performed in accordance with the terms of the German Animal Protection Law, as well as according to international animal welfare legislation and institutional ethical guidelines of the Charité Berlin, Germany, and were approved by the Landesamt für Gesundheit und Soziales (Approval number G 0175/13, LAGeSo Berlin, Germany).

In our chronic siRNA study, 8-week-old male C57BL6/J mice (Forschungseinrichtungen für experimentelle Medizin, Charité Berlin, Germany) were fed ad libitum a Western diet (WD) consisting of a high-fat diet (60% kcal from fat, D12492 (I), Ssniff Spezialdiäten, Soest, Germany) supplemented with 6% sucrose in the water for 7 weeks to induce insulin resistance and hepatic lipid accumulation.

In the first in vivo pilot study 8-week-old male C57BL6/J mice were fed a normal chow diet (ND) and were injected for 2, 4 or 6 days with 1 mg per kg body weight of either siRNA (siKeap1-1*, siKeap1-2* and siControl*) to assess efficacy and duration of siRNA action. In the second in vivo pilot study 8-week-old male C57BL6/J mice were fed a ND or a WD for 20 weeks.

All mice were maintained in individually ventilated cages (max. 5 per cage) in an environmentally controlled room with a 12 h light-dark cycle. Body weight was monitored weekly. For the siRNA intervention, mice were divided into three groups with equal body weight and fat mass. Each group was injected weekly with 1 mg modified siRNA per kg body weight in PBS of liver-selective Keap1-specific siRNA (group 1: siKeap1-1*, group 2: siKeap1-2*) or unspecific scrambled control siRNA (group 3: siControl*) via the tail vein for 7 weeks. Body composition (BC) was assessed by ^1^H magnetic resonance spectroscopy using a Minispec LF50 Body Composition Analyzer (Bruker BioSpin, Billerica, USA) before starting the intervention, metabolic cage analysis (MCA) and sacrificing (Ex). Isoflurane inhalation (1.5%) was used for anesthesia during operation and mice were sacrificed by cervical dislocation.

#### Glucose tolerance test (ipGTT)

Mice were fasted overnight and basal blood glucose was measured before injecting an intraperitoneal glucose bolus of 1 g/kg body weight. Blood glucose was measured subsequently at 0, 15, 30, 60 and 120 min.

#### Activity and calorimetric analysis

Various phenotypic parameters were assessed in metabolic cages with a TSE LabMaster System (TSE Systems, Bad Homburg, Germany). Mice were acclimated to special water bottles for 24 h and to the metabolic cages for 16 h before starting the measurements. Mice were supplied with WD, individually housed and data on gas exchanges, activity, and food/liquid intake were collected for 48 h. Calorimetry was performed with a computer-controlled open circuit calorimetry system composed of 10 metabolic/respiratory cages. Each cage was equipped with a special water bottle and a food tray connected to a balance as well as an activity monitor. Oxygen consumption and carbon dioxide production were measured for each mouse at 3 min intervals and respiratory quotient (RQ) was calculated as the ratio of CO_2_ production to O_2_ consumption. O_2_ consumption, CO_2_ production, energy expenditure (EE) and caloric intake were adjusted for lean body mass from the body composition measurements [23]. Data collected after 48 h were analyzed as (daily) average.

### siRNA Preparation

#### Synthesis, purification and formulation of liver-selective Keap1-specific siRNAs

RNA oligonucleotides were synthesized at a scale of 1 μmol (in vitro) or 10 μmol (in vivo) on an ABI 394 DNA/RNA or BioAutomation MerMade 12 synthesizer using commercially available 5′-O-DMT-3′-O-(2-cyanoethyl-N,N-diisopropyl) phosphoramidite monomers (SAFC) of uridine, 4-N-acetylcytidine (CAc), 6-N-benzoyladenosine (ABz) and 2-N-isobutyrylguanosine (GiBu) with 2′-O-TBDMS protection or 2’-OMe modification and 5′-O-DMT-thymidine-3′-O-(2-cyanoethyl-N,N-diisopropyl) phosphoramidite (dT) following standard protocols for solid-phase synthesis and deprotection [24, 25]. The crude oligonucleotides were analyzed by IEX and LC–MS and purified by anion-exchange high-performance liquid chromatography (IEX) using a linear gradient of 10–50% buffer B in 30min. Solutions of 0.02 M Na_2_HPO_4_ (pH 11) and 0.02 M Na_2_HPO_4_/1 M NaBr (pH 11) were used as eluents A and B, respectively. To ensure high fidelity of the data, all single strands were HPLC purified to > 85% purity. The purity and identity of the oligonucleotides was confirmed by ion-exchange chromatography and LC–MS, respectively. For the cell culture experiments, 100 μM stock solutions of siRNA in PBS were prepared by mixing equimolar amounts of complementary sense and antisense strands in 1x PBS buffer and heating the solution to 90°C for 10 min and allowing it to cool slowly to room temperature to complete the annealing process. siRNAs were further characterized by HPLC and were stored frozen until use. Sequences of the unmodified siRNAs directed against murine Keap1 mRNA (siKeap1-1 and siKeap1-2) and non-silencing scrambled control siRNA (siControl) are depicted in Table 1.

**Table 1:** Sequences of siRNA molecules used in this study.

siRNA	Sequence
siControl	5’-UUU CGC GUA UAC GCG AAA C dTdT-3’
siControl sense	5’-AUC GUA CGU ACC GUC GUA U dTdT-3’
siControl antisense	5’-AUA CGA CGG UAC GUA CGA U dTdT-3’
siKeap1-1 sense	5’-GCG CCA AUG UUG ACA CGG A dTsdT-3’
siKeap1-1 antisense	5’-UCC GUG UCA ACA UUG GCG C dTsdT-3’
siKeap1-2 sense	5’-CCU GCA ACU CGG UGA UCA A dTsdT-3’
siKeap1-2 antisense	5’-UUG AUC ACC GAG UUG CAG G dAsdA-3’

Capital letters: RNA; d: DNA.

Large scale RNA oligonucleotide synthesis for in vivo administration was carried out by Biospring (Frankfurt, Germany). Formulation of siRNA for delivery to mouse liver tissue in vivo was performed in lipid nanoparticles (LNPs) based on the Axolabs’ proprietary cationic lipid XL-10 (Axolabs, Kulmbach, Germany).

#### siRNA stability in mouse serum

Primary and modified siRNAs were tested for nuclease stability in 50% mouse serum. For this 160 μl of 2.5 μM siRNA in 1x DPBS and 160 μl mouse serum (Sigma) were incubated at 37°C for 0, 0.5, 2, 4, 6, 8 and 24 h. At each time-point 20 μl of the reaction mixture was removed and quenched with a stop solution (Lysis Solution, Proteinase K, water) at 65°C for 30 min. prior to HPLC analysis on a Waters 2695 Separation Module and a 2487 Dual Absorbance Detector, RNase-free water was added to each sample. The solution was analyzed by HPLC using a DNAPac PA200 analytical column (Thermo Scientific). Serum half-lives were estimated for both strands of the siRNAs and are shown in Table 2.

**Table 2:** Chemical modification of Keap1-specific siRNAs improved their pharmacokinetic properties.

siRNA	siKeap1-1	siKeap1-1*	siKeap1-2	siKeap1-2*
	(186.1)	(190.2)	(191.1)	(185.4)
**IC**_**50KD**_ (nM)	0.14	0.25	0.61	0.02
**I**_**maxKD**_ (%)	96	91	97	94
**Serum stability** (h):	> 4	> 8	2	4
t_1/2_ in 50% mouse serum				
**Cytotoxicity** (%):	85	96	89	89
Viability of Hepa1c1c7 cells				
**Immune stimulation** (pg/ml):	423	221	523	144
IFNα release PBMCs				

I_maxKD_: maximal knockdown of Keap1 mRNA expression

* modified siRNA.

#### Immune stimulatory potential of siRNA

Immuno-stimulative potential of siRNA was measured by secretion of IFNα from freshly isolated and transfected human peripheral blood mononuclear cells (PBMC). PBMCs were isolated from approx. 16 ml blood of three healthy donors that was collected in Vacutainer tubes coated with sodium heparin (BD, Heidelberg, Germany) according to the manufacturer’s instructions. Subsequently, 100 nM siRNA was reverse transfected into 100,000 PBMCs with 0.3 μl Lipofectamine 2000 per 96-well (n = 2) in a total volume of 150 μl serum-free RPMI medium for 24 h. Single-stranded RNA (“R-0006”) and DNA (“CpG ODN”) oligonucleotides were applied as positive controls. 25 μl of the supernatant was used for measurement of IFNα concentration using a self-established electrochemiluminescence assay based on MesoScale Discovery’s (MSD, Rockville, USA) technology. IFNα values are shown in Table 2.

#### siRNA transfection and in vitro validation

Hepa1c1c7 cells were reversely transfected with siRNAs using Lipofectamine RNAiMAX (Thermo Scientific) according to the manufacturer’s instructions. To avoid variations in the transfection efficiency when transfecting very low amounts for IC_50_ curves, 10 nM of non-targeting control (siControl*) were used in each transfection mix. The transfection was performed in 96-well plates adding 12,000 cells/well. 48 h post-transfection, RNA was isolated with the SV96 Total RNA isolation system and gene expression was analyzed as described, previously [26].

#### Cytotoxicity assay

Hepa1c1c7 mouse hepatoma cells were reversely transfected with siRNAs using Lipofectamine RNAiMAX according to the manufacturer’s instructions in 96-well plates. 72 h post-transfection, cell viability was analyzed with the ViaLight™ Plus Cell Proliferation and Cytotoxicity BioAssay Kit (Lonza, Cologne, Germany). Cytotoxicity was assessed in relation to the non-transfected control cells set at 100% (Table 2).

### Biochemical analysis

Plasma glucose levels of mice were determined by measuring at least in duplicate (≤ 10% discrepancy) using Contour XT glucometer (Bayer Vital, Leverkusen, Germany). Plasma lipid measurements were carried out with a Cobas 6000 c501 module (Roche/Hitachi) using the corresponding Cobas substrate kits according to the manufacturer’s instructions. Liver lipids were analyzed after lipid extraction described elsewhere [27, 28], with an AU680 Analyzer (Beckman Coulter/Olympus, Krefeld, Germany) and the corresponding kits. As an exception, phospholipids in serum and liver tissue were measured with a Phospholipid kit from DiaSys (Holzheim, Germany). Liver transaminases (AST, ALT) were measured in serum after the siRNA treatment. Liver glycogen content was measured on a xMark Microplate absorbance spectrophotometer (Bio-Rad, Hercules, USA) according to the method from bio-protocol [29].

### Histochemistry

Liver slices were snap-frozen, embedded in Tissue-Tek (O.C.T. Compound, Sakura, Staufen, Germany) on dry ice and 5 μm cryosections were prepared for oil red O (ORO) and H&E staining. ORO staining was carried out with 5 min incubation as described [30]. H&E sections were stained with Mayer`s Hemalaun for 15 min, 5 min blued, dipped 5x in activated Eosin-G-solution, drained in an ascending ethanol series and mounted with Roti-Histokit t II. Three sections per liver as technical replicates were analyzed in bright field with 20x magnification on a BZ-9000 microscope (Keyence, Neu-Isenburg, Germany) and images were quantified using ImageJ [31] according to nature protocols [30]. Representative images of each treatment group are depicted. Scale: black bars represent 50 μm.

### mRNA isolation and quantitative real-time PCR

Total RNA was extracted from snap-frozen tissue samples using TRIzol reagent according to the manufacturer’s protocol. 1 μg RNA was digested with DNase and transcribed into cDNA via RevertAid Reverse Transcriptase. Quantitative real-time PCR was performed on a Lightcycler 480 System (Roche). Relative quantification was performed using the ΔΔct method and expression data were normalized to Rpl37a gene expression. Following primer sets were used: Keap1 (Mm00497268_m1), NAD(P)H dehydrogenase, quinone 1 (Nqo1, Mm00500821-m1), glutamatecysteine ligase, catalytic subunit (Gclc, Mm00802655-m1), glutathione reductase (Gsr, Mm00439154_m1), NF-E2-related factor 2 (Nrf2, Mm00477784_m1), glutathione S-transferase A2 (Gsta2, Mm03019257_g1), Gsta4 (Mm004974803_m1), thioredoxin reductase 1 (Txnrd1, Mm00443675_m1), glutathione S-transferase mu 1 (Gstm1, Mm00833915_g1), Gstm2 (Mm00725711_s1), Gstm3 (Mm00833923_m1), Gstm4 (Mm00728197_s1), sulfiredoxin 1 (Srxn1, Mm00769566_m1), microsomal glutathione S-transferase (Mgst3, Mm00787806_s1), and glutathione peroxidase 2 (Gpx2, Mm00850074_g1), glyceraldehyde-3-phosphate dehydrogenase (Gapdh, Mm03302249_g1) and ribosomal protein L37a (Rpl37a, Mm01546394_s1) as TaqMan® Gene Expression Assay (Thermo Scientific).

### Microarray transcript expression profiling

Total RNA was isolated from liver samples of 4 fed and 4 fasted mice for each siRNA group. The integrity of RNA samples was controlled by RNA nano assay (Agilent 2100 BioAnalyzer) and transferred to Atlas Biolabs (Berlin) for analysis. Amplification, labeling of probes, Affymetrix GeneChip hybridizations, comparative microarray analysis, and subsequent statistical analysis were performed using Mouse Genome 430 2.0 GeneChips (Affymetrix), as described previously [21]. All Affymetrix microarray data have been deposited in MIAME compliant format at NCBI-GEO under the accession number GSE80956.

### Data and statistical analysis

If not indicated otherwise, all data are presented as mean ± SEM. GraphPad Prism 6 (La Jolla, USA) was used to calculate all statistics and to draw graphs. Statistical differences were calculated using one-way or two-way (repeated measurement) ANOVA or Kruskal-Wallis test depending on whether data passed normal distribution tests adjusted for multiple testing with Bonferroni’s multiple comparisons test (Bonferroni’s mct) for post hoc analysis where appropriate. P values less than 0.05 were considered significant.

## Results

### Structure, modification and evaluation of two liver-selective Keap1-specific siRNAs

**Fig 1:**
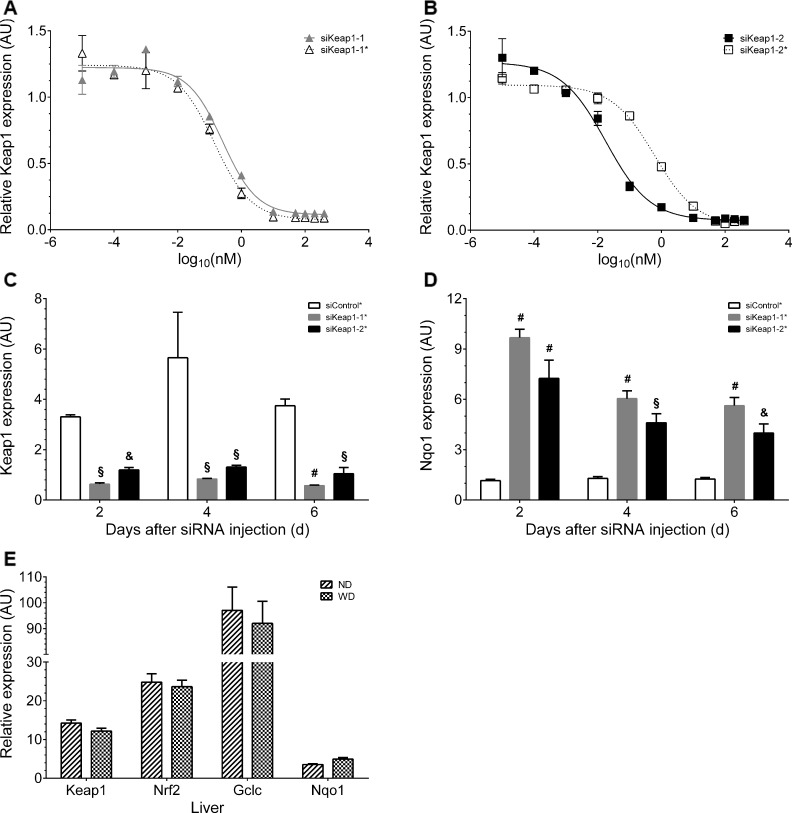
In vitro and in vivo validation of Keap1 knockdown by Keap1-specific siRNAs. (A-B) Dose-response curves upon transfection of Hepa1c1c7 cells with Keap1-specific siRNA molecules. Relative Keap1 expression inhibited by (A) the modified siKeap1-1* siRNA and its unmodified form (siKeap1-1) and (B) the modified siKeap1-2* siRNA and its unmodified form (siKeap1-2) (n = 2). (C-D) Gene expression of (C) Keap1 and (D) a Nrf2 target gene Nqo1 in mouse liver 2, 4 and 6 days after siRNA injection for a pilot study (n = 3). (E) Regulation of Keap1, Nrf2 and its target genes Gclc and Nqo1 in liver of mice fed either a normal chow diet (ND) or a Western diet (WD) for 20 weeks (n = 6). Data are represented as mean ± SEM. &: siRNA vs. siControl* p < 0.05, §: siRNA vs. siControl* p < 0.01, #: siRNA vs. siControl* p < 0.001. Two-way ANOVA with Bonferroni’s mct. siKeap1-1* and siKeap1-2*: modified Keap1-specific siRNAs, siControl*: modified scrambled unspecific control siRNA.

Recently, we have characterized two siRNAs (Table 1) against murine Keap1, siKeap1-1 and siKeap1-2, which are able to reduce Keap1 mRNA levels leading to activation of Nrf2 [21]. In the present study these siRNAs were modified by introducing 2’-O-methyl groups and phosphorothioate to allow chronic in vivo administration. The modified siRNAs, siKeap1-1* and siKeap1-2*, showed improved serum stability and a strongly reduced immune stimulatory potential in relation to the unmodified siRNAs (Table 2), while their potency to suppress Keap1 expression in mouse hepatoma cells remained unchanged (Fig 1A and 1B). For in vivo application the siRNAs were formulated with lipid nanoparticles that lead to a targeting of the oligonucleotides mainly to hepatocytes [32]. After single i.v. administration both Keap1-specific siRNAs showed an effective knockdown of hepatic Keap1 mRNA expression, which did not differ between days 2, 4 and 6 after the application. A modified non-silencing scrambled control siRNA (siControl*) did not alter Keap1 inhibition (Fig 1C). At day 6 siKeap1-1* and siKeap1-2* reduced Keap1 mRNA levels by 85% and 72%, respectively. In order to test, whether the knockdown of Keap1 led to an activation of Nrf2, the expression levels of the Nrf2 target gene Nqo1 (NAD(P)H quinone oxidoreductase 1) were determined. Hepatic Nqo1 transcript levels were elevated at day 2, 4 and 6 after either siKeap1-1* or siKeap1-2* injection with a 4.5- and 3.2-fold increase, respectively, at day 6 (Fig 1D). From this data it was concluded that a once weekly i.v. injection of either siKeap1-1* or siKeap1-2* was sufficient to both reduce Keap1 expression and to stimulate Nrf2-related gene expression in vivo. In a second pilot study, mice were fed either a WD or a normal chow diet (ND) for over 20 weeks. Neither a reduction of Keap1 mRNA expression nor an induction of Nrf2 or its target genes Gclc and Nqo1 could by observed in the WD group compared to the ND group (Fig 1E). Therefore, we did not include a ND control group in our chronic siRNA study.

### Gene expression analysis after chronic suppression of Keap1

**Fig 2:**
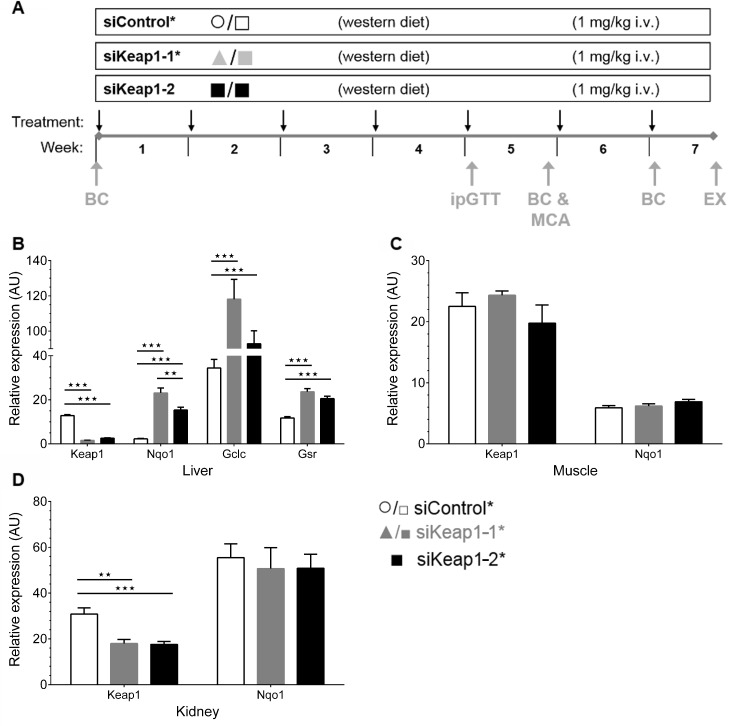
Keap1 inhibition and Nrf2 target gene regulation by liver-selective, Keap1-specific siRNAs in mice after siRNA and WD intervention. (A) Schematic of the long-term mouse experiment. (B) Hepatic Keap1 as well as Nrf2 target genes Nqo1, Gclc and Gsr expression (n = 11). (C) Relative mRNA expression of Keap1 and Nqo1 in muscle tissue of mice (fed, n = 6). (D) Expression levels of Keap1 and Nqo1 in kidney (fed, n = 6,). Data are represented as mean ± SEM. One-way ANOVA with Bonferroni's mct. ** p < 0.01, *** p < 0.001. Black arrows: Recording of body weight and siRNA injection, Grey arrows: Experimental procedures: BC: Analysis of body composition by ^1^H-nuclear magnetic resonance, EX: Sacrificing mice and organ preparation, ipGTT: intraperitoneal glucose tolerance test, MCA: Metabolic cage analysis.

In order to study the effect of chronic Keap1 knockdown, body weight-matched mice on a WD were treated once weekly with either siKeap1-1*, siKeap1-2 or siControl* for 7 weeks (Fig 2A). At the end of the experiment mRNA levels of Keap1 and selected Nrf2-target genes levels were determined in liver, skeletal muscle and kidney by RT-qPCR (Fig 2B–2D). After 7 weeks of intervention the hepatic Keap1 mRNA was significantly reduced by 88% and 80% for siKeap1-1* and siKeap1-2* siRNA, respectively (Fig 2B). The expression of the Nrf2-target gene Nqo1 increased 10.3- and 6.8-fold in response to siKeap1-1* and siKeap1-2*, respectively. In addition, the Nrf2-target genes glutathione reductase (Gsr) and glutamate cysteine ligase catalytic subunit (Gclc) were upregulated 2- and 1.8 fold as well as 3.4- and 2.7-fold for siKeap1-1* and siKeap1-2*, respectively. Neither siKeap1-1* nor siKeap1-2* influenced the expression of Keap1 or Nqo1 in muscle (Fig 2C). In the kidney siKeap1-1* and siKeap1-2* reduced the expression of Keap1 by 58% and 57% for siKeap1-1* and siKeap1-2*, respectively (Fig 2D). However, Nqo1 expression was not upregulated indicating that Nrf2-signaling was not significantly activated in kidney. Overall, the data suggest that the chronic administration of either siKeap1-1* or siKeap1-2* caused a regulation of Keap1 and Nrf2 target genes predominantly in liver.

**Fig 3:**
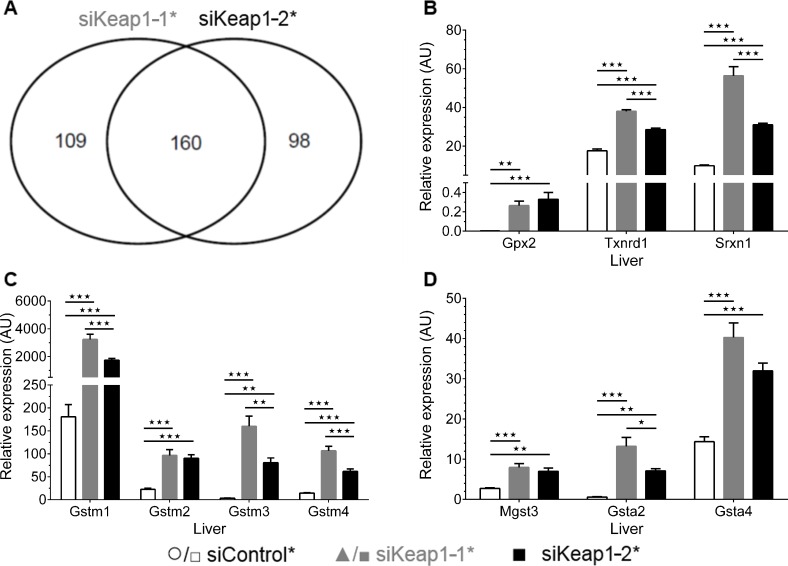
Overlap of probe sets regulated by siKeap1-1* and siKeap1-2* and verification of upregulated genes by quantitative real-time PCR. (A) Venn diagram of analyzed genes that were regulated by the administration of either siKeap1-1* or siKeap1-2* in relation to non-silencing siControl* (fold-change >2, two-way ANOVA p < 0.01, n = 8). (B-D) Relative gene expression of upregulated genes under siKeap1-1* and siKeap1-2* treatment in liver of mice (fed, n = 6). Data are represented as mean ± SEM. One-way ANOVA with Bonferroni's mct. * p < 0.05, ** p < 0.01, *** p < 0.001.

Whole genome expression analysis revealed that the chronic suppression of Keap1 by siKeap1-1* and siKeap1-2* regulated 269 and 252 probe sets, respectively, in relation to siControl* (S1 and S2 Tables). 160 probe sets were regulated by both siKeap1-1* and siKeap1-2* (Fig 3A and S3 Table). Mapping of this core set of probe sets regulated by both siRNAs to canonical pathways revealed the activation of pathways that are linked to cytoprotection and metabolism of xenobiotics (Table 3). The majority of these genes upregulated by both Keap1-specific siRNAs were also examined by quantitative real-time PCR to confirm the whole genome expression analysis and, therefore, the induction of Nrf2 target genes in the different oxidative stress response pathways (Fig 3B–3D) These pathways are characteristic for Nrf2 signaling [3, 33] and demonstrated that both siKeap1-1* and siKeap1-2* activated Nrf2 signaling chronically in liver. As metabolic pathway, only canonical cholesterol synthesis was down-regulated by both siRNAs against Keap1 (Table 3). Neither the core set of probe sets regulated by both siRNAs nor those that were regulated by only one of the siRNAs included any genes encoding proteins known to be involved in fatty acid- and glucose-handling (S1 and S2 Tables). Altogether, the gene expression data showed that the chronic administration of either siKeap1-1* or siKeap1-2* suppressed hepatic Keap1 expression and stimulated Nrf2-signaling in liver, but had no effect on genes encoding proteins that are involved in the metabolism of either fatty acids or carbohydrates.

**Table 3:** Canonical pathways regulated by both siKeap1-1* and siKeap1-2*.

Canonical pathway	Statistical significance	Regulated genes
Glutathione-mediated detoxification	8.1	Gsta1, Gsta2, Gsta4, Gstm1, Gstm2, Gstm3, Gstm4, Mgst3
Nrf2-mediated oxidative stress response	8.0	Gpx2, Gclc, Nqo1, Txnrd1, Srxn1
Superpathway of cholesterol biosynthesis	5.4	Hmgcr, Lss, Sc5d, Fads1, Fdps, Sc5d
Xenobiotic metabolism pathway	5.2	Cyp2b9, Cyp2b10, Cyp2g1, Cyp2a4, Gsta1, Gsta2, Gsta4, Gstm1, Gstm2, Gstm3, Gstm4, Mgst3

Genes significantly regulated in liver of mice by silencing Keap1 expression by two different Keap1-specific siRNAs versus siControl* were mapped by Ingenuity Pathway Analysis. The significance of the pathway is given as -log p value (Fisher's exact test).

### Chronic hepatic Keap1 knockdown affected neither body composition, weight gain nor the metabolic profile

**Fig 4:**
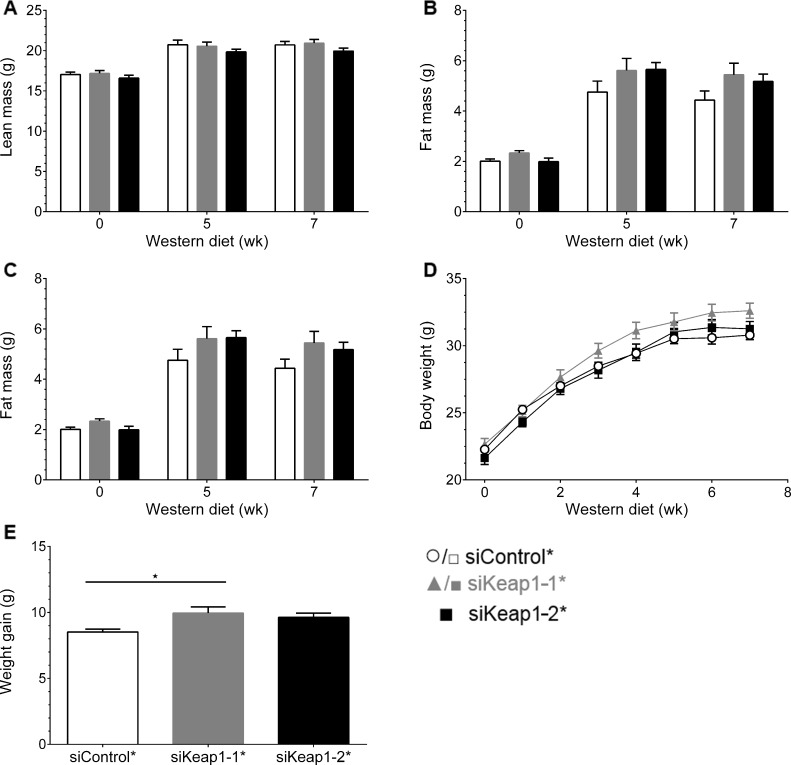
Body composition of mice during 7 weeks of WD and siRNA intervention. (A) Lean body mass, (B) free fluid and (C) fat body mass at beginning (0 weeks), in week 5 and at end of intervention (week 7) of mice treated with control and both Keap1-specific siRNAs. (D) Development of body weight under specific diet within 7 weeks. (E) Absolute body weight gain of each treatment group (* p = 0.015, one-way ANOVA with Bonferroni's mct). Data are represented as mean ± SEM. n = 11.

The effects of Keap1 suppression and the subsequent regulation of Nrf2 target genes on body composition were assessed at day 0 and in weeks 5 and 7 before sacrificing (S4 Table). Treatment with neither siKeap1-2* nor siKeap1-2* affected lean body mass, free body fluids, body-fat content and weight gain relative to siControl* (Fig 4A–4D). Absolute body weight gain was slightly increased 1.2-fold in the siKeap1-1* group relative to siControl*; siKeap1-2* had no significant effect (Fig 4E). Hepatic Keap1 suppression influenced neither whole-body fat accumulation nor weight gain.

**Fig 5:**
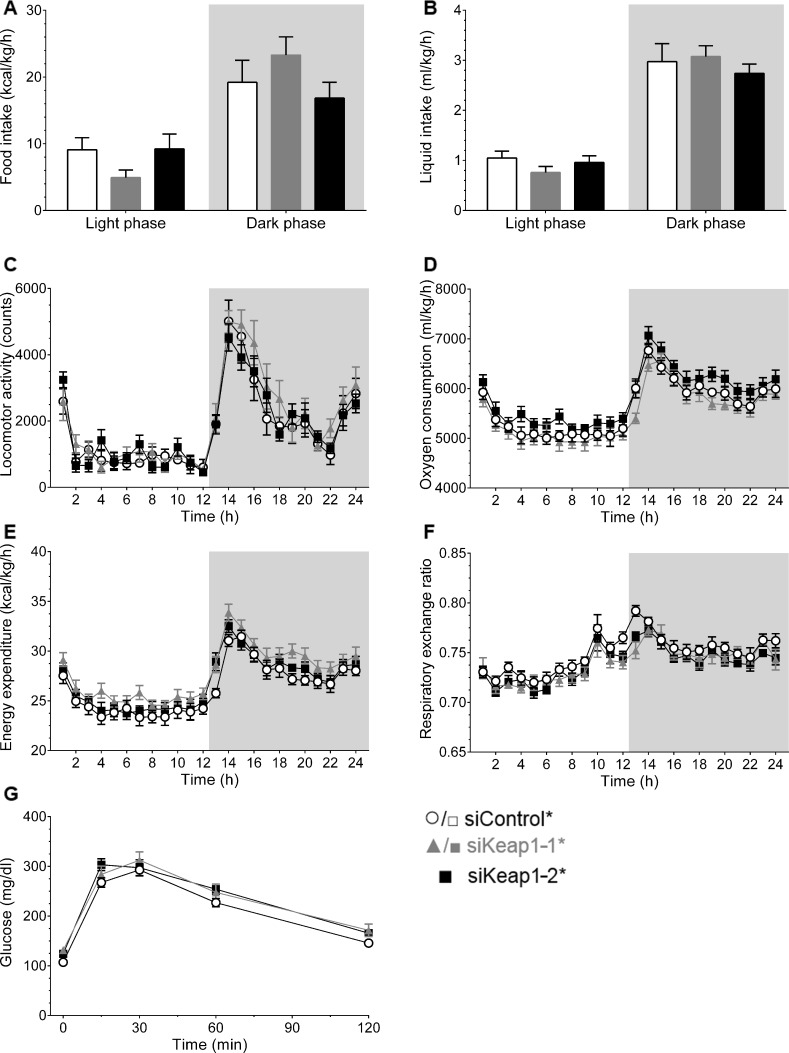
Metabolic characterization of siRNA-treated mice. (A-F) 24 h average of metabolic cage analysis during light and dark phase of (A) food intake, (B) water consumption supplemented with 6% sucrose, (C) locomotor activity, (D) oxygen consumption rate, (E) energy expenditure and (F) respiratory exchange ratio (VCO_2_/VO_2_). (siControl* n = 9, siKeap1-1* n = 9 and siKeap1-2* n = 10). (G) Blood glucose levels (glucose: 1 mg/kg BW) during ipGTT (n = 11). Data are represented as mean ± SEM. Grey background in graphs indicates dark phase.

A metabolic cage study was performed after 5 weeks of intervention. Neither siKeap1-1* nor siKeap1-2* affected food and water intake over 24 h (Fig 5A and 5B). The total caloric intake combined from the HFD and the 6% sucrose in the drinking water was comparable (Table 4). Furthermore, the locomotor activity during day and night time followed natural habits with a rapid increase at onset of dark phase and was similar between all groups (Fig 5C). The oxygen consumption (VO_2_) and the according carbon dioxide production (VCO_2_) showed no differences between the treatment groups in relation to animals treated with siControl* (Fig 5D and Table 4). In addition, the energy expenditure (EE) adjusted on lean body mass and the respiratory exchange ratio (RER), as ratio of VCO_2_ and VO_2_, revealed no effect of reduced Keap1 expression on basal metabolism (Fig 5E and 5F). Also in week 5 an ipGTT was performed in order to assess a potential impact of hepatic Keap1 knockdown on glucose homeostasis. The administration of either siKeap1-1* or siKeap1-2* revealed no effect on glucose tolerance (Fig 5G).

**Table 4:** Basal metabolic characterization of siRNA-treated mice.

siRNA	siControl*			siKeap1-1*			siKeap1-2*		
Locomotor activity (counts/h)	1721	±	132	1983	±	137	1770	±	95
Oxygen consumption (ml/kg/h)^A^	5613	±	104	5505	±	125	5832	±	127
Carbon dioxide production (ml/kg/h)^A^	4213	±	62	4083	±	73	4321	±	92
Energy expenditure (kcal/kg/h)^A^	26.8	±	0.5	26.2	±	0.6	27.8	±	0.6
Total caloric intake (kcal/kg/h)	29.2	±	4.0	29.1	±	2.9	26.9	±	3.5

Mice fed a WD and received a liver-selective siRNA treatment. Data are represented as mean ± SEM. n = 9/9/10. ^A^values calculated on lean body mass.

**Fig 6:**
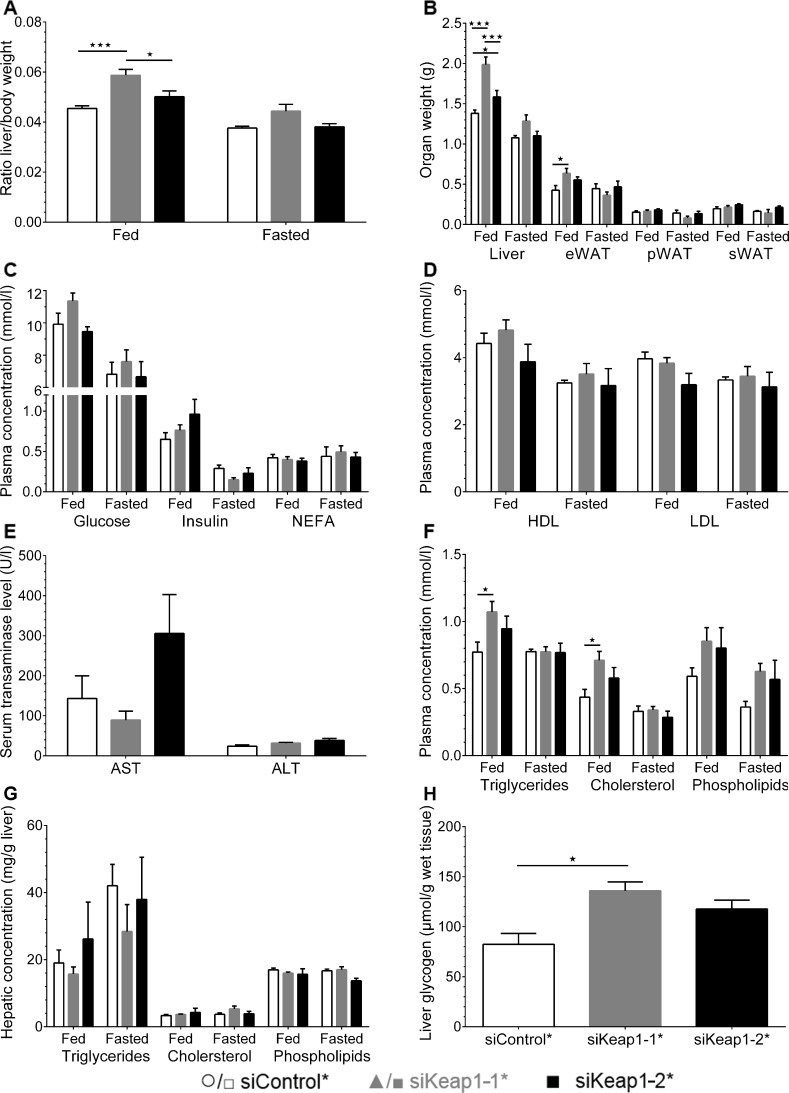
Plasma and hepatic parameter of mice either fed or fasted for 16 h under specific siRNA treatment. (A) Ratio of liver weight to body weight. (B) Weight of liver and epididymal (e), perirenal (p) and subcutaneous (s) white adipose tissue (WAT). (C) Basal blood glucose, insulin and non-esterified fatty acids (NEFA) concentration. (D) Plasma levels of HDL and LDL cholesterol. (E) Concentration of enzymes AST (aspartate transaminase) and ALT (alanine transaminase) in serum of fed mice. (F-G) Triglyceride, cholesterol and phospholipid concentration in (F) plasma and (G) liver lysates. (H) Glycogen concentration in liver tissue of fed mice. Data are represented as mean ± SEM. Fed: n = 6, fasted: n = 5. Two-way ANOVA with Bonferroni's mct. * p < 0.05, *** p < 0.001.

**Fig 7:**
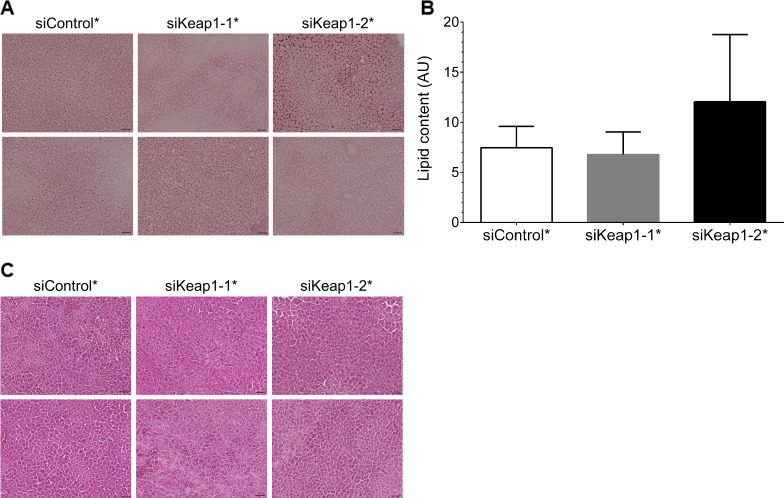
Oil Red O (ORO) and H&E stainings of fed mice treated with Keap1-specific siRNAs. (A) Neutral lipids stained by ORO in liver section. (B) Quantification of ORO stainings by densitometric analysis (fed, n = 6). (C) H&E staining of liver sections. Representative images are depicted in A and C. Data represent mean ± SEM. n = 6. Black scales indicate 50 μm.

After the treatment with siKeap1-1*, but not with siKeap1-2* the liver / body weight ratio and the liver weight of fed animals were slightly elevated (Fig 6A and 6B). Epididymal WAT weight was slightly increased by siKeap1-1*. Neither siKeap1-1* nor siKeap1-2* influenced significantly plasma glucose levels, insulin or NEFA levels in the fed or fasted state (Fig 6C). HDL and LDL levels were also not significantly different between the treatment groups (Fig 6D). Additionally, concentrations of the liver enzymes AST (aspartate transaminase) and ALT (alanine transaminase) were also measured in serum of fed mice. However, no evidence for increased hepatic inflammation mediated by either the WD or hepatotoxicity induced due to the siRNA administration was revealed (Fig 6E). The administration of siKeap1-1* slightly elevated plasma levels of triglycerides and cholesterol in fed state (Fig 6F). Neither siKeap1-1* nor siKeap1-2* affected hepatic lipids (Fig 6G). As we observed an increased liver weight and plasma triglyceride content but no elevated triglyceride levels in liver of siKeap1-1* treated mice, we additionally analyzed the hepatic glycogen content of fed mice. The glycogen concentration in the liver was again significantly increased under siKeap1-1* but only slightly elevated under siKeap1-2* siRNA treatment (Fig 6H). Therefore, we analyzed liver sections of fed mice by Oil Red O (ORO) staining to further verify the hepatic content of neutral lipids. However, we did not observe an altered amount or a modified size of lipid droplets in either Keap1-specific siRNA group compared to the control group (Fig 7A). The densitometric quantification of neutral lipids stained by ORO showed also no significant differences between each group (Fig 7B) In a last step, we monitored liver morphology using H&E stainings and did not reveal obvious pathological morbidities in liver tissues of any treatment group (Fig 7C).

Considering the number of endpoints analyzed, we found no conclusive differences on any of the investigated metabolic traits.

## Discussion

In this study we investigated the effects of chronic activation of Nrf2 signaling in liver on gene expression and metabolic parameters in mice fed on a WD for 7 weeks. Nrf2 signaling was activated by the suppression of Keap1 as described previously [22]. Here, we used two different liver-selective siRNAs against Keap1 in order to reduce the risk of unspecific off-target effects. In liver, the target organ of the modified siRNAs, a strong suppression of Keap1 expression was accomplished by both Keap1-specific siRNAs and subsequent activation of Nrf2 target genes was observed in liver but not in kidney. Notably, we observed a slight yet significant downregulation of Keap1 mRNA in kidney of siKeap1-1* and siKeap1-2*-treated mice. This did not result in a significant upregulation of the prototypical Nrf2 target gene Nqo1, most likely due to the relatively moderate downregulation of Keap1 in kidney.

Whole genome expression analysis and canonical pathway mapping proved the regulation of canonical Nrf2 signaling in liver by the siRNAs. As expected genes involved in antioxidative stress response and xenobiotic metabolism were induced by the suppression of Keap1. This is in line with a previous report using a constitutive model of Keap1 suppression [33]. However, neither genes involved in the metabolism of fatty acids nor of carbohydrates were regulated by the activation of Nrf2. In line with our gene expression results we could not detect alterations of metabolic parameters such as hepatic and serum triglyceride levels or glucose tolerance. Interestingly, we observed a suppression of genes involved in the synthesis of cholesterol, although both plasma and hepatic cholesterol levels were unchanged. As the inhibition of cholesterol synthesis by e.g. statins has only a minor effect in mice, future studies will have to investigate more appropriate animal models of cholesterol metabolism, such as guinea pigs, in order to elucidate the potential of Nrf2 activation in the regulation of cholesterol levels. Although the effects of siKeap1-1* and siKeap1-2* were similar, they slightly differed with respect to their action on weight gain and the liver / body weight ratio in the fed state as well as hepatic triglyceride and glycogen content which were expected to be reduced under Keap-1-specific siRNA treatment. It is unclear, if these discrepancies are due to either off-target effects or to the slightly higher suppression of Keap1 expression by siKeap1-1*. However, in the latter case the increased body weight gain does also not indicate that increased activation of Nrf2 is a viable approach for the treatment of metabolic disease. Our data differ from some previous publications, in which metabolic effects as well as changes in the gene expression of e.g. fatty acid synthase 1, stearoyl-CoA desaturase 1, acetyl-CoA carboxylase and glucose 6-phosphatase catalytic subunit were described after genetic or pharmacological activation of Nrf2 [6–8, 11, 16, 34–36]. The pharmacological small-molecule Nrf2 activators such as CDDO and Oltipraz have been associated with toxicity in chronic studies [9, 10], which will impact intermediary metabolism and could be a confounding factor in previous studies. Major differences between our studies and previous genetic models are the timing and tissue selectivity of the genetic activation of Nrf2. Previously, the effects of genetic Nrf2 activation were studied in floxed Keap1 mice [16, 35–37], making use of the hypomorphic character of the floxed allele, which leads to a constitutive expression of Nrf2 in multiple tissues [38]. The hepatocyte-specific Keap1 knockout animals used in [11] were generated by breeding these Keap1 floxed mice with albumin-Cre transgenic mice. [38]. With it in previous genetic models the effects of a life-long, whole-body activation of Nrf2 were studied. In contrast, we activated Nrf2 expression in liver of adult mice using siRNAs. This approach eliminates potential developmental consequences of the constitutive, whole-body activation of Nrf2 expression. We believe that our approach to activate Nrf2 in adult animals may more appropriately reflect the therapeutic situation and the lack of effects under those conditions raises doubts whether hepatic Nrf2 activation is a feasible therapeutic target.

In our study, mice were fed a WD for 7 weeks. We found no evidence that HFD/WD per se induces oxidative stress or an induction of Nrf2. This was demonstrated in a pilot study feeding mice either a normal chow diet (ND) or a WD over 20 weeks. Within that study we did not observe a downregulation of Keap1 nor an upregulation of Nrf2 or its target genes Nqo1 or Gclc in WD-fed compared to ND. Therefore we did not include a ND group in our chronic study. However, under certain artificial dietary conditions (such as a methionine- and choline-deficient diet) pronounced oxidative or inflammatory stress might have been induced which could stimulate Nrf2. The WD showed only a mild increase in body weight gain and liver lipids (e.g. triglycerides, glycogen, neutral lipids, ORO stainings) in all treatment groups and both Keap1-specific siRNAs showed no consistent benefit of siRNA treatment on WD feeding. In contrast to our expectations, we did not observe a decrease in weight gain or hepatic triglyceride accumulation. We cannot exclude that the activation of Nrf2 in a model of increased oxidative stress may have indirect metabolic effects through the activation of anti-inflammatory or antioxidative pathways.

In summary, our results show that the chronic suppression of Keap1 expression in liver stimulates Nrf2 target genes involved in antioxidative stress response and xenobiotic metabolism, but has no significant effect on hepatic lipid and glucose metabolism in mice fed a WD. Hence, beneficial effects on glucose metabolism and lipid handling reported by others may result from an extra-hepatic, not liver-intrinsic Nrf2 activation [6–8, 11, 34–36]. With that we found no evidence that the activation of hepatic Nrf2 could be a target for the treatment of features of metabolic diseases, such as hepatic insulin resistance or steatosis.

## Supporting Information

S1 Table Probe sets regulated by siKeap1-1*. (XLSX)

S2 Table Probe sets regulated by siKeap1-2*. (XLSX)

S3 Table Probe sets regulated by both siKeap1-1* and siKeap1-2*. (XLSX)

S4 Table Body weight and composition of siRNA-treated mice. (DOCX)
