# Correction: Chronic Activation of Hepatic Nrf2 Has No Major Effect on Fatty Acid and Glucose Metabolism in Adult Mice

**DOI:** 10.1371/journal.pone.0172657

**Published:** 2017-02-16

**Authors:** Sebastian Brachs, Angelika F. Winkel, James Polack, Hui Tang, Maria Brachs, Daniel Margerie, Bodo Brunner, Kerstin Jahn-Hofmann, Hartmut Ruetten, Joachim Spranger, Dieter Schmoll

There is an error in Panel A of Fig 3. Fig 3A should show the number 92 instead of 98 for genes only regulated by siKeap1-2*. Please see the correct Fig 3 and its caption below.

**Fig 3:**
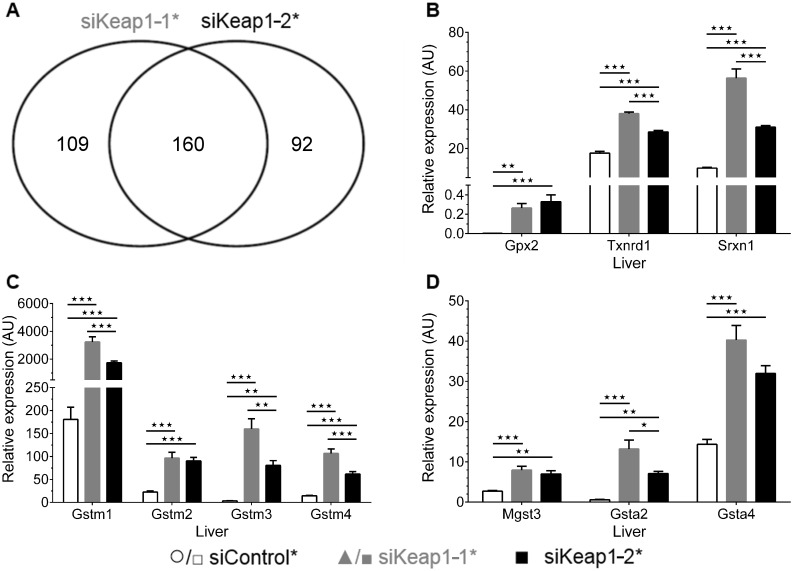
Overlap of probe sets regulated by siKeap1-1* and siKeap1-2* and verification of upregulated genes by quantitative real-time PCR. (A) Venn diagram of analyzed genes that were regulated by the administration of either siKeap1-1* or siKeap1-2* in relation to non-silencing siControl* (fold-change >2, two-way ANOVA p < 0.01, n = 8). (B-D) Relative gene expression of upregulated genes under siKeap1-1* and siKeap1-2* treatment in liver of mice (fed, n = 6). Data are represented as mean ± SEM. One-way ANOVA with Bonferroni's mct. * p < 0.05, ** p < 0.01, *** p < 0.001.

There is an error in Panel B of Fig 4. Fig 4B should show Free fluid instead of Fat mass. Please see the correct Fig 4 and its caption below.

**Fig 4:**
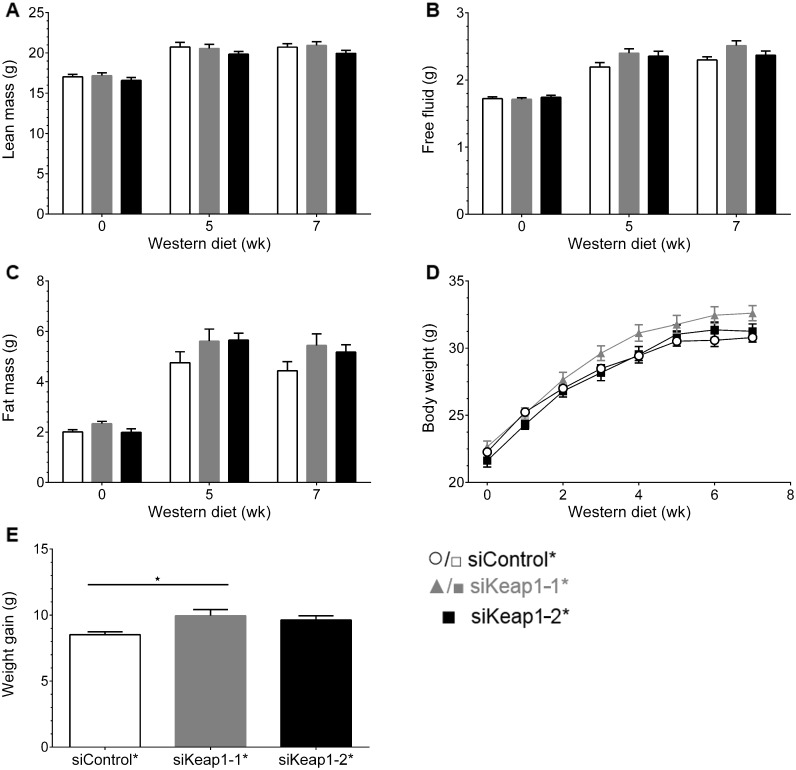
Body composition of mice during 7 weeks of WD and siRNA intervention. (A) Lean body mass, (B) free fluid and (C) fat body mass at beginning (0 weeks), in week 5 and at end of intervention (week 7) of mice treated with control and both Keap1-specific siRNAs. (D) Development of body weight under specific diet within 7 weeks. (E) Absolute body weight gain of each treatment group (* p = 0.015, one-way ANOVA with Bonferroni's mct). Data are represented as mean ± SEM. n = 11.
