# Supplementary material for: Chronic Activation of Hepatic Nrf2 Has No Major Effect on Fatty Acid and Glucose Metabolism in Adult Mice
Source: PLoS One. 2016 Nov 4;11(11):e0166110. doi: 10.1371/journal.pone.0166110 (PMC5096693; doi:10.1371/journal.pone.0166110)
Supplement: S4 Table — (DOCX) [file pone.0166110.s004.docx]

**S4 Table.** **Body weight and composition of siRNA-treated mice.**

| siRNA | | siControl* | | | siKeap1-1* | | | siKeap1-2* | | |
| --- | --- | --- | --- | --- | --- | --- | --- | --- | --- | --- |
| Starting body weight (g) | | 22.3 | ± | 0.3 | 22.7 | ± | 0.4 | 21.6 | ± | 0.5 |
|  | Fat mass (g) | 2.0 | ± | 0.1 | 2.3 | ± | 0.1 | 2.0 | ± | 0.1 |
|  | Lean body mass (g) | 17.0 | ± | 0.3 | 17.2 | ± | 0.4 | 16.6 | ± | 0.4 |
| Terminal body weight (g) | | 30.8 | ± | 0.3 | 32.6 | ± | 0.6^§^ | 31.3 | ± | 0.5 |
|  | Fat mass (g) | 4.4 | ± | 0.4 | 5.4 | ± | 0.5 | 5.2 | ± | 0.3 |
|  | Lean body mass (g) | 20.7 | ± | 0.4 | 20.9 | ± | 0.5 | 19.9 | ± | 0.4 |
| Total body weight gain (g) | | 8.5 | ± | 0.2 | 10.0 | ± | 0.5^§^ | 9.6 | ± | 0.3 |

Mice fed a Western diet and received a liver-selective siRNA treatment. Data are represented as mean ± SEM. n = 11. ^§^ siRNA vs. siControl* p < 0.05, one-way ANOVA with Bonferroni's mct.
